# Supplementary material for: Dynamic integration of forward planning and heuristic preferences during multiple goal pursuit
Source: PLoS Comput Biol. 2020 Feb 18;16(2):e1007685. doi: 10.1371/journal.pcbi.1007685 (PMC7048318; doi:10.1371/journal.pcbi.1007685)
Supplement: S1 Table — (PDF) [file pcbi.1007685.s001.pdf]

**Offer**	**Points**	**Response**	**Classification**
Ab	$Pts_t^A - Pts_t^B > 1$	accept	g1
Ab	$Pts_t^A - Pts_t^B > 1$	wait	g2
Ab	$Pts_t^A - Pts_t^B < -1$	accept	g2
Ab	$Pts_t^A - Pts_t^B < -1$	wait	g1
Ab	$Pts_t^A - Pts_t^B = 1$	accept	g1
Ab	$Pts_t^A - Pts_t^B = 1$	wait	g2
Ab	$Pts_t^A - Pts_t^B = -1$	accept	nan
Ab	$Pts_t^A - Pts_t^B = -1$	wait	nan
Ab	$Pts_t^A - Pts_t^B = 0$	accept	g1
Ab	$Pts_t^A - Pts_t^B = 0$	wait	g2
aB	$Pts_t^A - Pts_t^B > 1$	accept	g2
aB	$Pts_t^A - Pts_t^B > 1$	wait	g1
aB	$Pts_t^A - Pts_t^B < -1$	accept	g1
aB	$Pts_t^A - Pts_t^B < -1$	wait	g2
aB	$Pts_t^A - Pts_t^B = 1$	accept	nan
aB	$Pts_t^A - Pts_t^B = 1$	wait	nan
aB	$Pts_t^A - Pts_t^B = -1$	accept	g1
aB	$Pts_t^A - Pts_t^B = -1$	wait	g2
aB	$Pts_t^A - Pts_t^B = 0$	accept	g1
aB	$Pts_t^A - Pts_t^B = 0$	wait	g2
