# Supplementary figures and images for: Dynamic integration of forward planning and heuristic preferences during multiple goal pursuit
Source: PLoS Comput Biol. 2020 Feb 18;16(2):e1007685. doi: 10.1371/journal.pcbi.1007685 (PMC7048318; doi:10.1371/journal.pcbi.1007685)

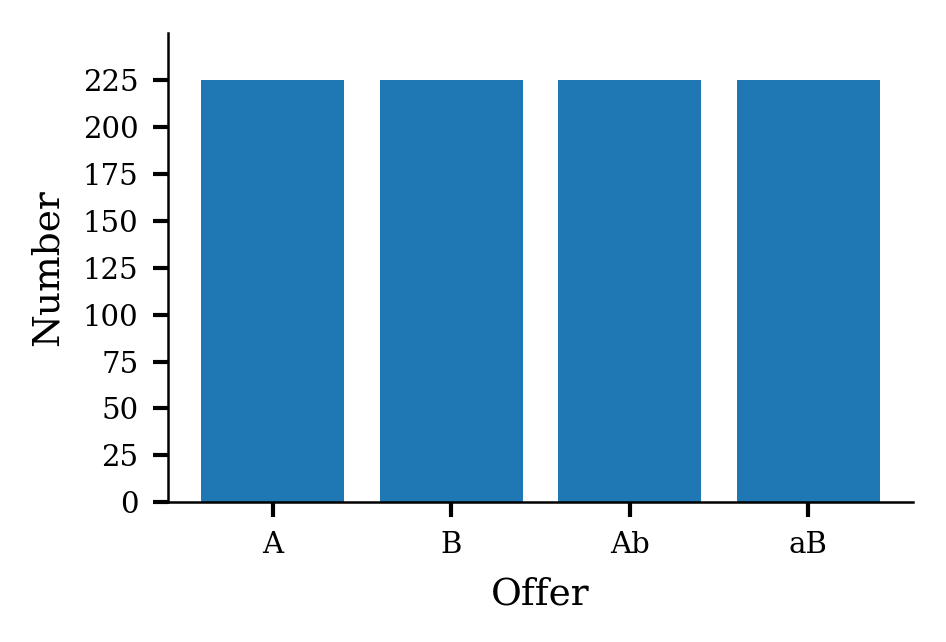

Supplement: S1 Fig — (PNG) [file pcbi.1007685.s002.png]

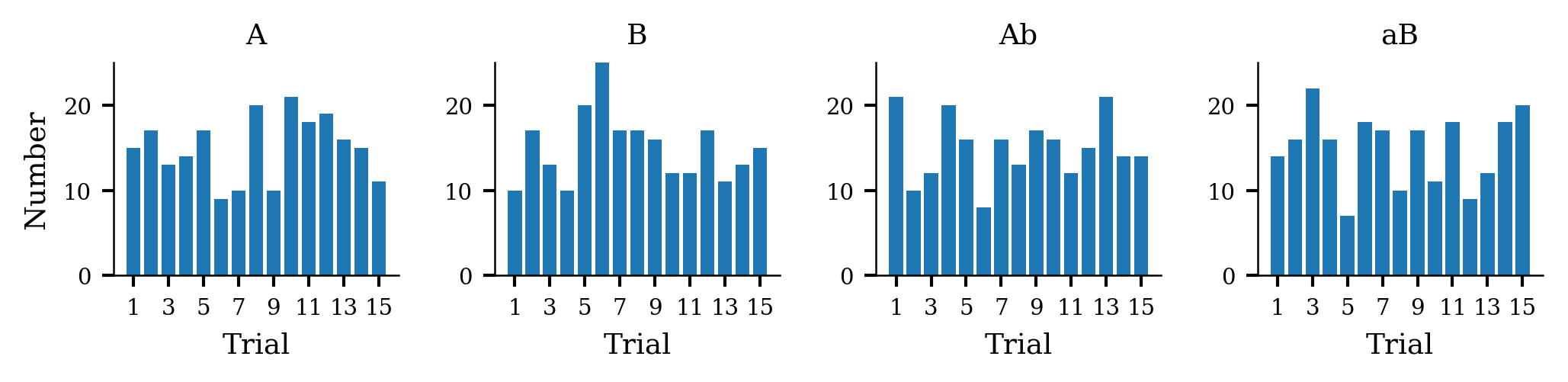

Supplement: S2 Fig — (PNG) [file pcbi.1007685.s003.png]

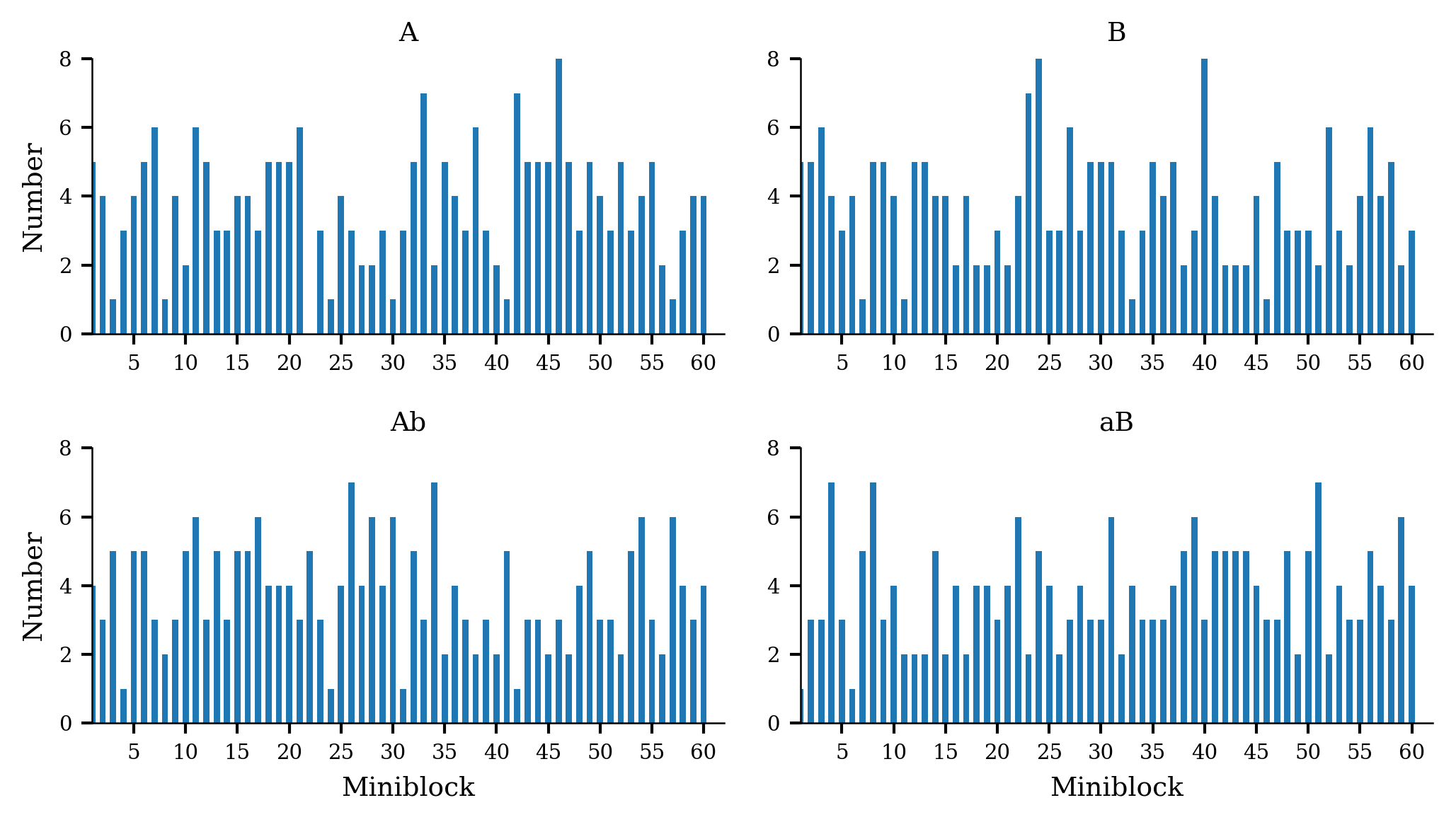

Supplement: S3 Fig — (PNG) [file pcbi.1007685.s004.png]

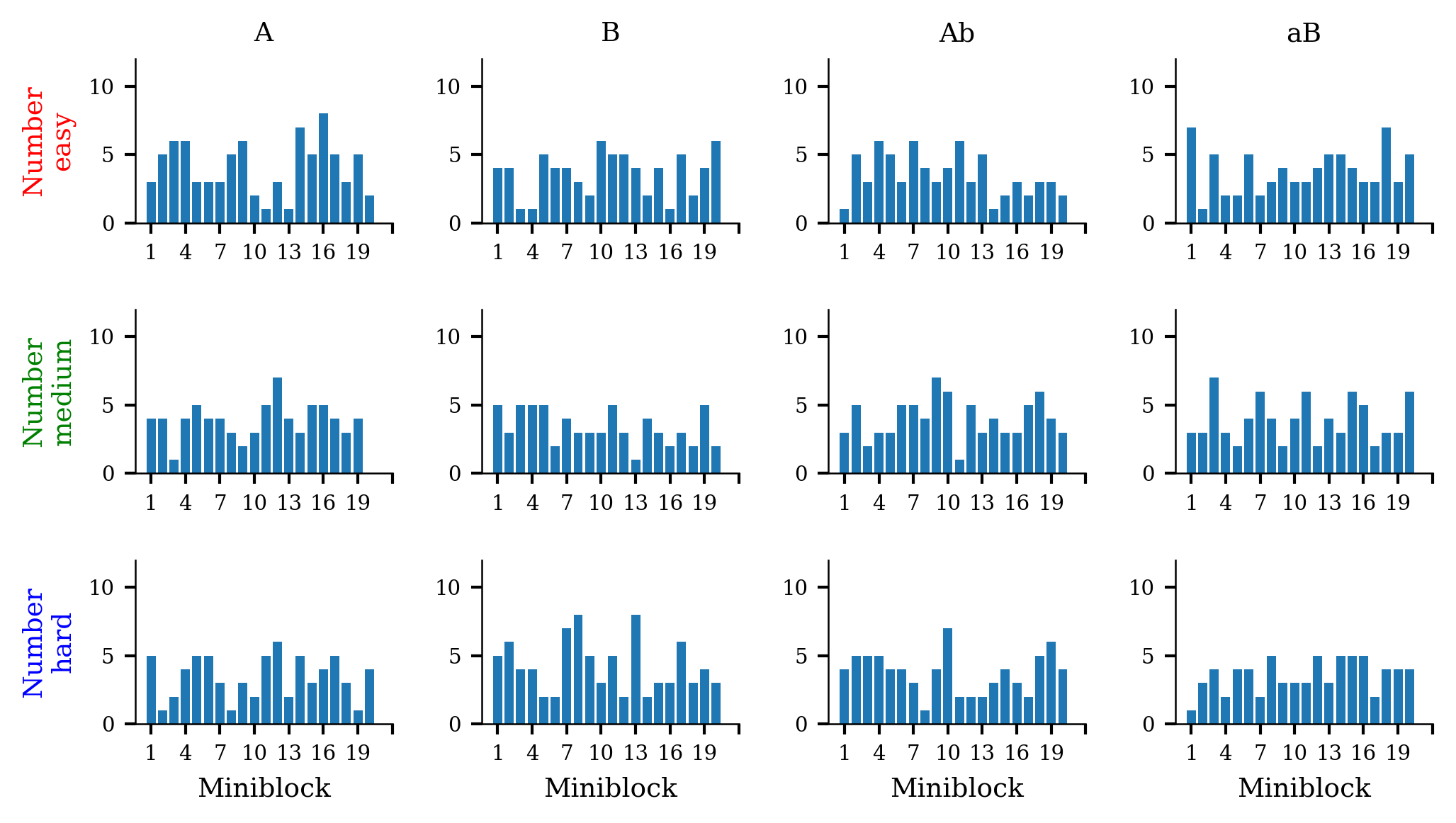

Supplement: S4 Fig — (PNG) [file pcbi.1007685.s005.png]

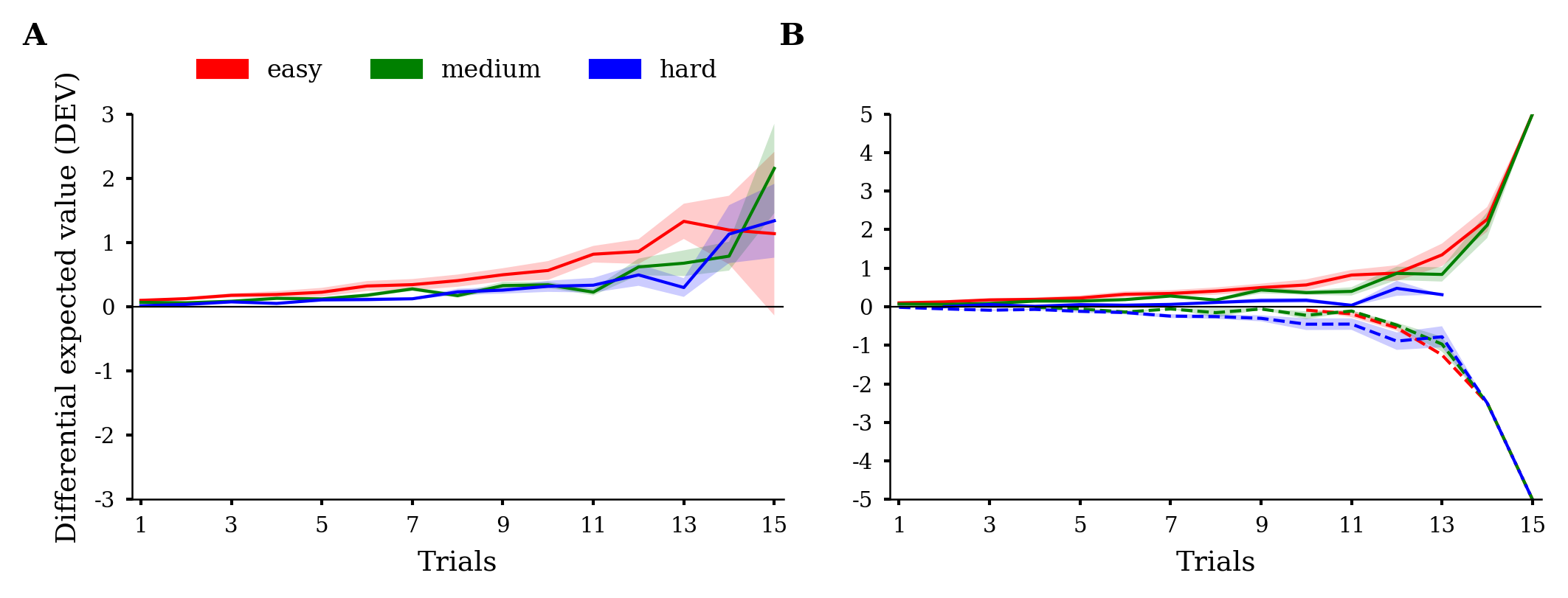

Supplement: S5 Fig — Average absolute (A) and signed (B) differential expected value (DEV) per trial and condition. Discount and reward ratio had been fixed (γ = 1, κ = 1). Average absolute DEVs at the beginning of the miniblock are smaller than in the end, indicating the relative importance of decisions close to the final trial of miniblocks. Conditions are colour coded. The shaded areas represent SD. (PNG) [file pcbi.1007685.s006.png]

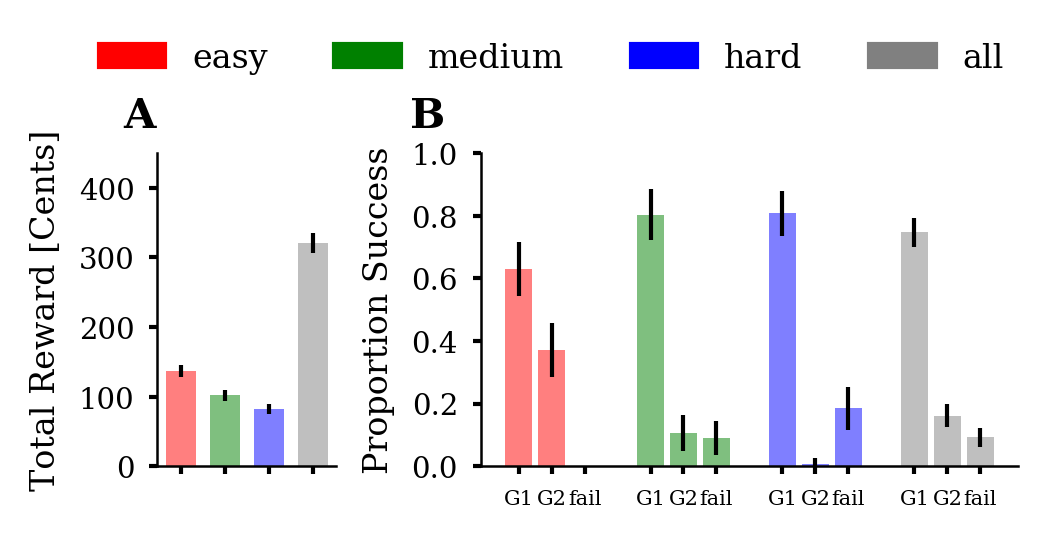

Supplement: S6 Fig — (A) Average total reward across agent instances (n = 1000). (B) Proportion of successful goal-reaching, averaged across agent instances, for each of the three conditions. We plot the proportion of reaching, at the end of a miniblock, a single goal (G1), both goals (G2), or no goal (fail). The random agent achieves fewer G2-successes in easy and medium than the participants but fails more often in medium and hard. The three conditions are colour-coded (easy = red, medium = green, blue = hard) and the average over conditions is shown in grey. Error bars depict SD. (PNG) [file pcbi.1007685.s007.png]

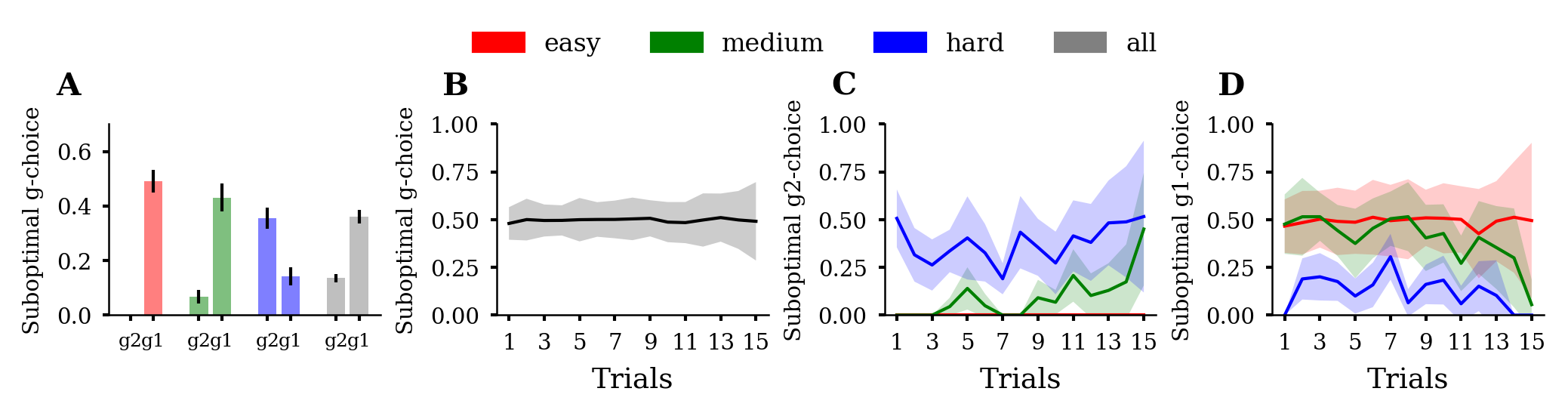

Supplement: S7 Fig — (A) Proportions of suboptimal g1-choices (g1) and suboptimal g2-choices (g2), averaged over agent instances (n = 1000). The random agent makes many suboptimal g1-choices in the easy and medium and many suboptimal g2-choices in the hard conditions. Summing together g1 and g2 yields approximately 50% suboptimal g-choices. (B) Suboptimal g-choices as a function of trial averaged over agent instances. The random agent makes approximately 50% suboptimal g-choices across all trials in the miniblock. If participants use non-random response strategies, i.e. planning or heuristics, their pattern of suboptimality across trials should deviate from the straight-line pattern of the random agent. (C) Suboptimal g2-choices as a function of trial averaged over agent instances. (D) Suboptimal g1-choices as a function of trial averaged over agent instances. Summing together g1 (D) and g2 (C) yields approximately 50% suboptimal g-choices across trials. Error bars and shaded areas depict SD. Conditions are colour coded. (PNG) [file pcbi.1007685.s008.png]

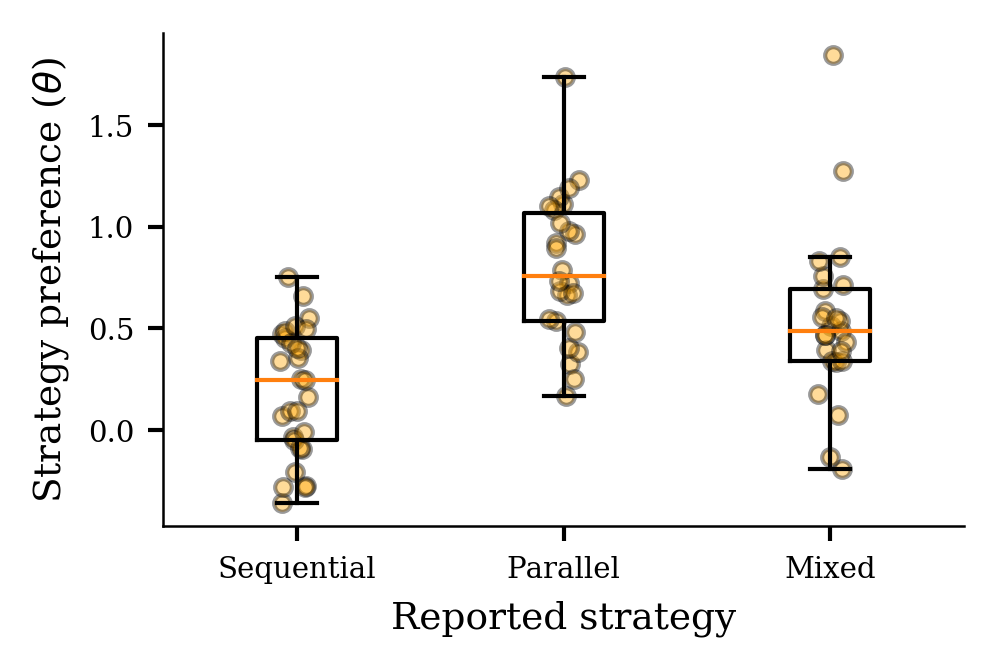

Supplement: S8 Fig — Participants who reported the use of a sequential strategy had lower estimated strategy preference, including the most negative values, than participants who reported the use of a parallel strategy. Participants who reported mixed use of a parallel and sequential strategy had greater strategy preference than the sequential group but lower estimates than the parallel group. The plot shows 80 of 89 participants whose verbal reports matched with one of the three strategy categories. (PNG) [file pcbi.1007685.s009.png]

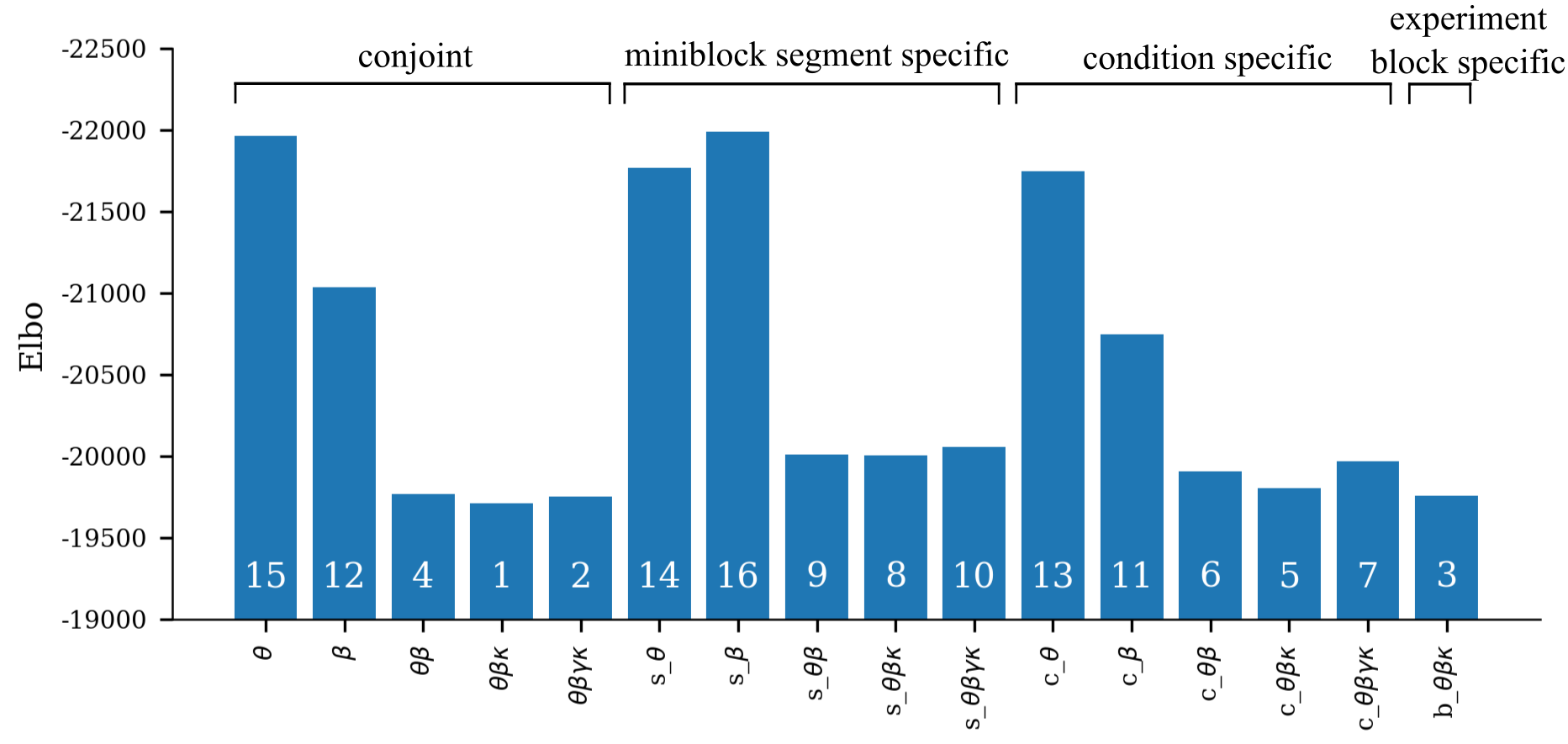

Supplement: S9 Fig — White numbers represent the rank from highest to lowest Elbo. Model comparisons showed that the three parameter model (θ,β,κ) had the highest model evidence. Adding γ did not increase model evidence (elboθβκ−elboθβγκ = −44). Estimating model parameters separately for miniblock segments (trial 1–5, trial 6–10, trial 11–15; prefix ‘s_’ in the figure) had lower model evidence compared to the winning model (elboθβκ−elbos_θβκ = −294). Estimating model parameters separately for conditions (easy, medium, hard; prefix ‘c’ in the figure) had lower model evidence compared to the winning model (elboθβκ−elboc_θβκ = −94). Estimating model parameters separately for experiment blocks (miniblock 1–20, miniblock 21–40, miniblock 41–60; prefix ‘b’ in the figure) had also lower model evidence compared to the winning model (elboθβκ−elbos_θβκ = −48). Bars in the plot depict Elbo averaged over the last 20 posterior samples. (PNG) [file pcbi.1007685.s010.png]

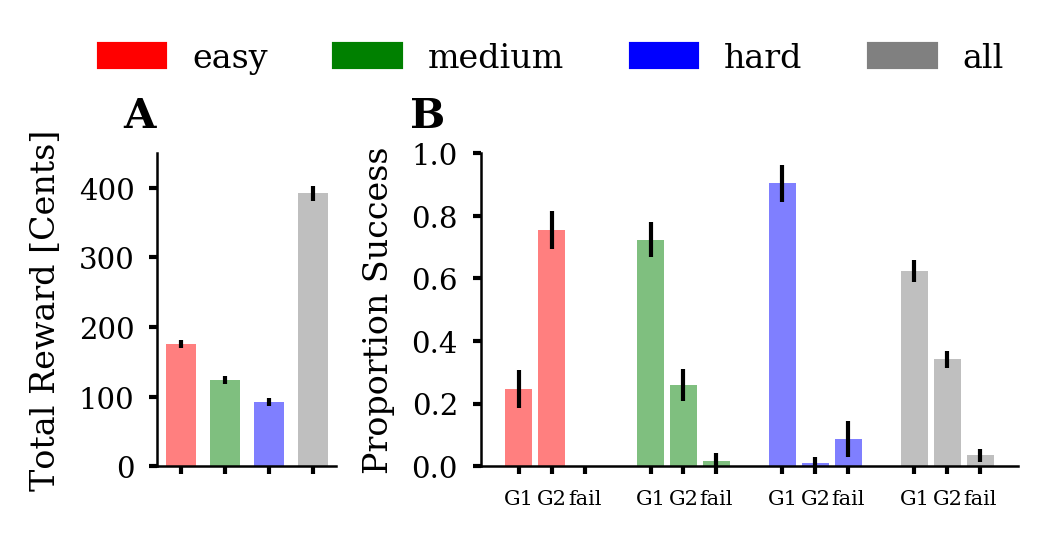

Supplement: S10 Fig — (A) Average total reward across samples (n = 1,000). (B) Proportion of successful goal-reaching, averaged across samples, for each of the three conditions. We plot the proportion of reaching, at the end of a miniblock, a single goal (G1), both goals (G2), or no goal (fail). The three conditions are colour-coded (easy = red, medium = green, blue = hard) and the average over conditions is shown in grey. Error bars depict SD. Data were generated using 1,000 posterior samples from the group hyper parameters. (PNG) [file pcbi.1007685.s011.png]

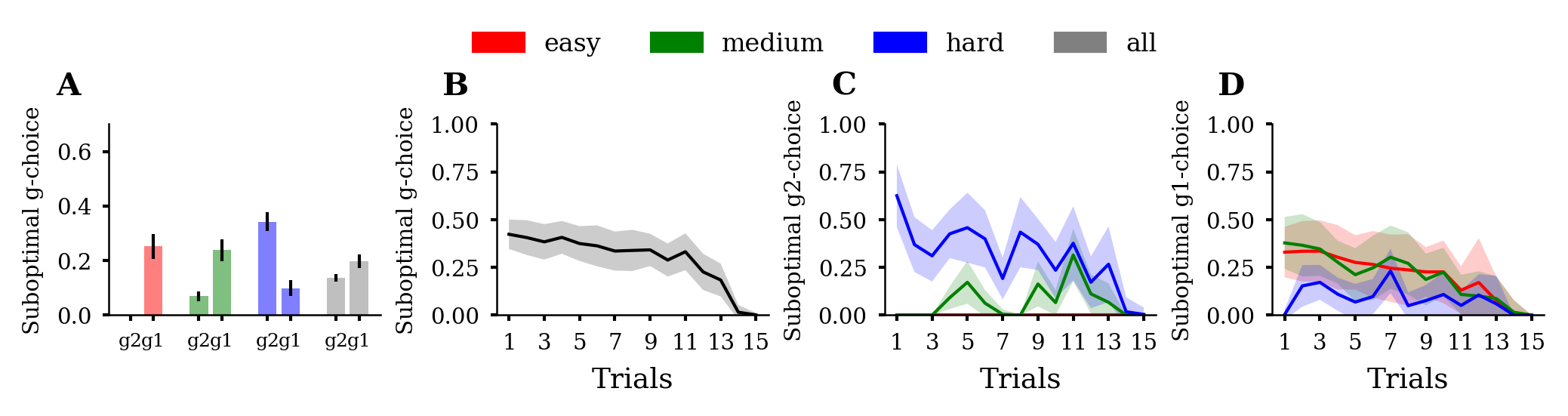

Supplement: S11 Fig — (A) Proportions of suboptimal g1-choices (g1) and suboptimal g2-choices (g2), averaged over samples (n = 1,000). (B) Suboptimal g-choices as a function of trial averaged over samples. (C) Suboptimal g2-choices as a function of trial averaged over samples. (D) Suboptimal g1-choices as a function of trial averaged over samples. Error bars and shaded areas depict SD. Conditions are colour coded. Data were generated using 1,000 posterior samples from the group hyper parameters. (PNG) [file pcbi.1007685.s012.png]

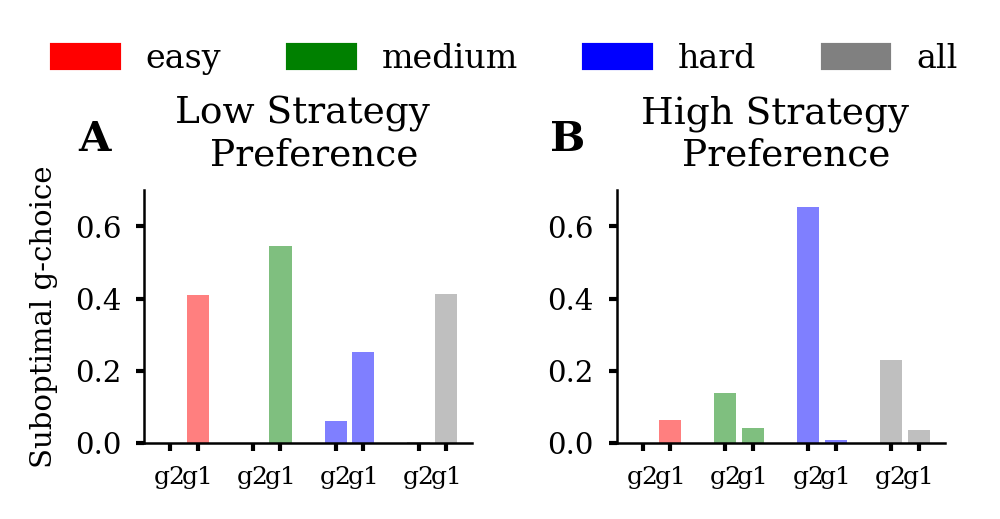

Supplement: S12 Fig — The plot shows proportions of suboptimal g1-choices (g1) and suboptimal g2-choices (g2) (A) of the participant with the lowest fitted strategy preference (θ = −0.36) and (B) of the participant with the highest fitted strategy preference (θ = 1.84). The low strategy preference participant prefers a sequential strategy leading to suboptimal g1-choices in the easy and medium condition. The participant with a high strategy preference parameter prefers a parallel strategy, resulting in a few suboptimal g1-choices in easy in and medium but a large number of suboptimal g2-choices in the hard condition. (PNG) [file pcbi.1007685.s013.png]
