# Supplementary material for: Dynamic integration of forward planning and heuristic preferences during multiple goal pursuit
Source: PLoS Comput Biol. 2020 Feb 18;16(2):e1007685. doi: 10.1371/journal.pcbi.1007685 (PMC7048318; doi:10.1371/journal.pcbi.1007685)
Supplement: S1 Text — (PDF) [file pcbi.1007685.s014.pdf]

Dear participant,
your task in this experiment is to reach goals. Within a block, consisting of 15 trials, you can either reach goal A, goal B or both goals at the same time. For one reached goal you will gain additional 5 Cents and for two reached goals additional 10 Cents. Your task is to obtain as much money as possible.

To reach goals, you must collect points. You can get points by accepting an offer. Some offers however, might have a negative effect on the state of a goal. Your task is to decide in every trial, whether to accept an offer or wait for the next offer. Press “up arrow” to accept an offer and “down arrow” to wait.

Important: Please decide deliberately but speedily. If you decide too slowly, you will get a notification. After every 5 notifications, 50 Cents will be subtracted from your bonus-payout. (The experiment starts with a training phase, in which no money can be lost.)

More about the goals:
Your goal progress will be represented by a bar, which is labelled with A or B. A goal counts as achieved, if one of the bars reaches or surpasses the white horizontal mark. The goal state will be evaluated after the end of the 15 trials.

More about the offers:
There are 4 different offers – A, B, Ab an aB. All offers have the same occurrence probability of 25%. The offers differ with respect to their effect on the goal state. A increases the A-bar by one point. B increases the B-bar by one point. Ab increases the A-bar by one point and subtracts one point from the B-bar. aB increases the B-bar by one point and subtracts 1 point from the A-bar.

Initial conditions:
At the beginning of the block, you already have some A- and B-points. The amount of initial points varies from block to block.
